# Free healthcare for some, fee-paying for the rest: adaptive practices and ethical issues in rural communities in the district of Boulsa, Burkina Faso

**DOI:** 10.1080/11287462.2021.1966974

**Published:** 2021-08-13

**Authors:** Thomas Druetz, Alice Bila, Frank Bicaba, Cheick Tiendrebeogo, Abel Bicaba

**Affiliations:** a Department of Social and Preventive Medicine, School of Public Health, University of Montreal, Montreal, Canada; b Centre de recherche en santé publique, Montreal, Canada; c Center for Applied Malaria Research and Evaluation, Department of Tropical Medicine, Tulane University, New Orleans, LA, USA; d Société d’Études et de Recherche en Santé Publique (SERSAP), Ouagadougou, Burkina Faso

**Keywords:** Free healthcare, user fee abolition, ethical issues, incapacity to pay, deontological dilemma, Burkina Faso

## Abstract

In Burkina Faso, in July 2016, user fees were removed at all public healthcare facilities, but only for children under 60 months of age and for “mothers”, i.e. for reproductive care. This study was conducted in five rural communities in Boulsa District (Burkina Faso) (1) to understand the perceptions and practices of stakeholders regarding compliance with eligibility criteria for free care and (2) to explore the ethical tensions that may have resulted from this policy. Semi-directed individual interviews (*n *= 20) were conducted with healthcare personnel and mothers of young children. Interviews were recorded and transcribed, and a thematic content analysis was conducted. The study reveals the presence of practices to circumvent strict compliance with the eligibility criteria for free access. These include hiding the exact age of children over 60 months and using eligible persons for the benefit of others. These practices result from ethical and economic tensions experienced by the beneficiaries. They also raise dilemmas among healthcare providers, who have to enforce compliance with the eligibility criteria while realizing the households’ deprivation. Informal adjustments are introduced at the community level to reconcile the healthcare providers’ dissonance. Local reinvention mechanisms help in overcoming ethical tensions and in implementing the policy.

## Introduction

Despite significant progress over the past 20 years, maternal and child mortality remains a major public health problem in low- and moderate-income countries (United Nations Development Program, 2020). In Burkina Faso, a country ranked 182nd out of 189 on the Human Development Index, the under-5 mortality rate was estimated at 7600 per 100,000 live births in 2018, and the maternal mortality rate at 320 per 100,000 live births, compared to an average of 500 and 12 in high-income countries, respectively (Hug et al., 2019; WHO et al., 2015).

Although this heavy burden of mortality is caused by a set of interrelated factors from different spheres (economic, social, biological, environmental, etc.), it is systematically more important in rural and deprived populations (Black et al., 2016; Denno & Paul, 2017; Okwaraji & Edmond, 2012) with poverty and rurality acting as fundamental causes, to use Link and Phelan’s expression (Link & Phelan, 2002). The crux of the issue is that a large proportion of these maternal and child deaths could be prevented by primary care or standard therapeutic interventions (Claeson et al., 2003; Khan et al., 2006; Liu et al., 2015; Ng et al., 2014). Therefore, addressing the lack of access to high-quality primary healthcare is a priority of the Sustainable Development Goals, encapsulated in the Universal Health Coverage policy (Kieny et al., 2017; Pettigrew et al., 2015; Rutherford et al., 2010).

Burkina Faso has taken various measures to improve financial accessibility to maternal and child healthcare. As early as 2006, it introduced the policy of subsidizing emergency obstetric and neonatal care, which reduced the price of reproductive health services by 80% (Ganaba et al., 2016). Around the same time, a few pilot projects were initiated in certain districts to abolish direct payment for children under the age of five at health facilities (Ridde, 2014). Finally, in 2016, the country scaled up to the national level the exemption from direct payment in public health facilities for maternal and child care (Gouvernement du Burkina Faso, 2016). This user fee exemption covers the costs of consultations, diagnosis, prescribed drugs and transportation to a referral hospital. It applies to all children under the age of five, regardless of the reason for the consultation, and for reproductive care (pre- and postnatal consultations, deliveries, cesareans, etc.).

Scientific studies have shown, in Burkina Faso and elsewhere in sub-Saharan Africa, the positive impacts of free healthcare policies on population health. Such policies improve access to healthcare, decrease catastrophic health spending and health inequalities, shorten the time before consultation, and reduce self-treatment practices as well as the proportion of home deliveries (Bassani et al., 2013; De Allegri et al., 2012; Druetz et al., 2015; Dzakpasu et al., 2014; Hatt et al., 2013). Evidence also suggests that the abolition of direct payment improves certain morbidity indicators and reduces neonatal mortality (El-Khoury et al., 2012; McKinnon et al., 2015; Qin et al., 2018). Implementation studies reveal broad acceptability and support from communities and health personnel, in addition to debunking certain myths regarding their feasibility (Bicaba et al., 2020; Carasso et al., 2012; Druetz et al., 2017; Ridde et al., 2014; Tama et al., 2018). However, they also highlight pressures that can affect the quality of care, healthcare providers’ motivation, health system weaknesses, and the fundamental issue of sustainability (Ansu-Mensah et al., 2021; Brunet-Jailly, 2018; Witter, 2009).

Despite these numerous studies, little knowledge has been gathered on the ethical issues surrounding free policies. In particular, while some writings suggest that the eligibility criteria are sometimes difficult to meet and can even give rise to dilemmas or tensions in the community, this dimension remains unexplored by rigorous qualitative studies (Nimpagaritse & Bertone, 2011 ; Pardeshi, 2014). How do the actors perceive the limitations of the user fee exemption, and how have they appropriated this policy through their practices? This problem becomes even more important as evidence accumulates regarding the extent of the needs of certain categories of ineligible individuals, notably school-aged children (Clark et al., 2020). The objective of this study is twofold: (1) to understand the perceptions and practices of health personnel, beneficiaries, and caregivers with regard to compliance with the eligibility criteria for free healthcare and (2) to explore the ethical tensions that have arisen from it and the coping mechanisms settled at the community level.

## Methods

A cross-sectional qualitative study was carried out in the Boulsa health district in Burkina Faso to understand the ethical issues related to compliance with the eligibility criteria for free healthcare. We conducted semi-structured individual interviews with the main beneficiaries and providers of free care: mothers of young children (*n* = 10) and members of the healthcare staff (*n* = 10). A COREQ checklist (consolidated criteria for reporting qualitative research) is available in Additional File 1.

Interviews were first conducted with healthcare staff until saturation was reached. After reviewing personal notes and discussions between the interviewer and other team members, the interview guide for mothers was reworked. To balance the volume of information collected between the two categories of participants, ten more interviews were conducted with mothers; again, saturation had been reached. The collection took place between September and December 2018, while the implementation of the free service was in a routine phase (two years after its introduction). At the time of their enrolment, participants were unknown to the research team.

### Study site

This study was conducted in five rural communities in the Boulsa District: Niega, Bonam, Boala, Yarcé and Zambanga. The district was selected for convenience, as it is a rural district in a safe and accessible area where staff from the health district authorities were known and trusted by the research team, and where there had not been a pilot project of free healthcare before the introduction of the national policy in July 2016. The Boulsa District is located in the North-Central region, about 100 km from the capital, and has a population of approximately 210,000 served by 19 primary health centres (Ministère de la Santé, 2017). The five communities were purposively selected after consultation with the district’s authorities, based on the proximity to the nearest primary health centre with maternity services.

### Recruitment of participants

In each primary health centre, the head nurse was approached and, after briefly outlining the purpose of the study, we sought his/her consent. We then approached a dedicated maternal health staff member (one midwife or auxiliary midwife per health centre) for recruitment.

In each of the five communities, two households were purposefully identified using the assistance of the local community health worker. The eligibility criteria for the households were that they well established in the community (resided there for several years) and had at least one child under 5 years of age and one mother above 18 years old. In each household, the head was informed of the project and, upon approval, an adult mother of a child under five was approached to proceed with recruitment. All approached individuals agreed to participate and gave consent.

### Data collection

For the health staff, interviews were conducted at the health centre in a consultation room, while the mothers were interviewed in their homes. All interviews were individual and semi-structured, with an interview guide specific to the type of participant that was developed for this study (Additional File 2). Interviews were conducted in a room or a remote location that guaranteed the confidentiality of the respondents. The guide was slightly enriched as the data were collected, particularly with respect to sub-questions used to reopen the discussion or to explore a theme in greater depth. Interviews with caregivers lasted approximately 30 min, while those with mothers lasted between 30 and 60 min. They were all conducted by a single female interviewer with vast experience in qualitative research (A Bila) and who speak the local language.

Interviews were conducted in French or in Mooré, depending on the participant’s preference. They were recorded, transcribed verbatim and translated into French (in the case of those conducted in Mooré). The transcripts were validated by a second person who listened to the original audio recordings. Field notes were taken during the interviews and used during the transcripts’ validation to add non-verbal information to the material. Transcripts were not returned to participants.

### Analyses

A thematic content analysis was carried out on all the material (Miles et al., 2014). We developed a common coding grid and independently coded the transcripts line by line (Additional File 3). We identified the dominant categories within the collected data and defined them as themes. Initial themes were discussed with team members and reformulated as necessary; some emerging themes that were accepted as significant were added. Double coding was performed to ensure that the themes were understood in the same way. The rare discrepancies (*n *< 10) were discussed and reconciled after clarification. QDA Miner software was used to facilitate the analyses. The participants did not provide feedback on the findings.

### Ethical considerations

This study has been approved by the Ethics Committee for Health Research in Burkina Faso (Deliberation #2018-6-075) and has received research authorization from the Ministry of Health of Burkina Faso (#2018-3032/MS/SG/DGESS/DSS). All participants provided written consent after the researcher explained the objectives of the study, read the consent form, and ensured that the information was understood. The researcher also presented the research institutions and their role in the project. The audio recordings and transcripts were stored on a computer with secure access. Participation was not remunerated.

## Results

A total of 20 individual interviews were conducted, 10 with healthcare providers and 10 with mothers (as beneficiaries and caregivers). The main sociodemographic characteristics of the participants are summarized in Table 1. Most of the interviewed mothers (7/10) were illiterate and work at their homes (6/10). They all lived in small dwellings made of mud, which is common in rural Burkina Faso, and had no visible access to electricity.

**Table 1.** Sociodemographic characteristics of the participants.

		Caregivers (mothers)	Healthcare providers
Number of interviews		10	10
Type of household			
Monogamous		4	NA
Polygamous		6	NA
Average number of children [range]		4.4 [2–7]	NA
Average number of children <5 [range]		1.9 [1–3]	NA
Principal occupation of the participant			
Housewife		6	NA
Agriculture		2	NA
Market seller		2	NA
Level of education of the participant			
None		7	0
Primary school		3	0
Secondary school or higher		0	10
Position			
Birth attendant		NA	3
Midwife		NA	2
Head nurse		NA	5
Average number of years of experience [range]		NA	5.5 [1–10]

### Knowledge about the exemption’s eligibility criteria

All providers and beneficiaries are aware of the existence of the free healthcare policy. They all recognize that this policy applies to all pregnant women as well as to children from 0 to 5 years old. However, two ambiguities remain with regard to the target populations. On the one hand, mothers do not know exactly whether they are entitled to free postpartum care, nor until when; the official limit (free postpartum care up to 42 days after delivery) is difficult to comprehend. On the other hand, there is ambiguity regarding children, as some caregivers think that free care includes children aged 5 years, while others think that it excludes them and in fact only concerns children aged 0–59 months.

Also, some mothers claim that free care is universal in nature; i.e. it covers all types of care for the target population. Health personnel confirms this misinterpretation and point out that, according to the guidelines, free care is universal only for children aged 0–5 years, and not for pregnant women. For the latter, only specific and pregnancy-related care is free.

### Circumventing practices from beneficiaries

As mentioned above, lack of knowledge of the strict eligibility criteria leads to situations where a patient may be denied free care when they consider in good faith to be entitled to it. But there are also several deliberate circumventing practices, reported by both health personnel and mothers, to extend the benefits of free care to situations where it is not normally applied.

One of the most commonly reported practices is to hide the exact age of children aged 60 months or older in order to become eligible. This practice sometimes results in impersonation, when identification documents of another child under the age of 5 (e.g. younger sibling) are brought in as proof of age.

Often they bring the child, you can see that the child is over five years old, but she says that the child is not even five years old; they don’t bring the child’s notebook, so it’s difficult to define the child's age. (HP3)

[If] they bring the little brother’s health booklet and come and say it’s his name, and it's his age. We can’t know. (CG6)

Another example is using an eligible person to receive a free consultation or medication for the benefit of another accompanying family member.

The women who come to pick up the products, they’re likely going to give them to their husbands, their co-wives or their children who are a little bit older but no longer eligible. (HP7)

Many people do that, bring the little one to the clinic when the big one is sick. (CG5)

If the child’s age goes up, you know he/she can no longer benefit. You can take advantage of the paracetamol from the small child to give to the big one. (CG1)

Even if it’s not true, you’re going to sit down and close your eyes (laughs). And you hope that they give you the drug! And you take it to give it later to your children. (CG2)

These practices are risky, however, because the treatment given to one beneficiary is not necessarily the same as the treatment that another family member should have.

They don’t know the dosage here. If it’s a child under the age of five, if it’s a small child, for example, it’s one tablet twice a day, whereas if it’s a child who’s 6 or 7 years old, the child can’t take one tablet twice, it’s two tablets, twice a day. She doesn’t know. She’ll think if I take him there, I’ll pay, but I have nothing to pay, so I have to take the one for whom the products are free, get the products and give them for his big brother. Sometimes it’s not easy, she won’t tell you it’s to give to another child. (HP1)

Some providers also report that beneficiaries go to several health centres in order to accumulate a larger supply of drugs that can be used either to treat other family members or to build up a stockpile of drugs that can be used later.

Yes, some women here can go to several health centres, that is, they can get up and consult here in Boala and then go to Bonam. That’s how they do it anyway, some women do it to get a lot of medication. (CG6)

As we go along, we realize that there are people who come twice, three times a week or even people who change their CSPS [health centre] to get treatment to take the medication and give it to someone else. (HP2)

Sometimes this practice stems from the lack of medicines available in a particular health centre, which leads some beneficiaries to visit different centres.

Some of the circumventing practices are specific to women of childbearing age. For example, some women of childbearing age may take advantage of the free services when they are pregnant to deal with health problems that are not related to pregnancy, and some use the free visits for their young children as a pretext when what they really want is to receive confidential family planning advice.

Yes, a woman is going to take advantage of her pregnancy to cure other illnesses, her old illnesses … anyways. She takes advantage of pregnancy to receive care. To heal. (GC5)

To receive family planning, many [women] take advantage of free visits of their children to do so. (GC9)

### Motives of circumventing practices

The main reason put forward for wanting to go beyond the strict threshold for receiving free healthcare is directly related to economic vulnerability. This situation concerns most households, and especially women, as they do not have income-generating activities and generally do not have control over the household’s finances. Ultimately, the decision to visit a health centre is left to husbands. They are less inclined to spend on maternal and child healthcare than the mothers, who are the primary caregivers in the family. During the dry season and the hunger gap, households’ ability to spend is even more limited due to the absence of harvests.

It depends on the head of the household. If you want [to receive healthcare], but you have nothing and your husband is not going to give you anything, if you’re concerned, you’re going to argue and say that you’re going to look for a solution by lying about the age of the child. You look for a solution to … to get the medicine. (GC10)

Mothers argue that the free healthcare policy does not in itself raise any injustice since the same eligibility criteria apply to all households. However, the problem of access due to an inability to pay for healthcare shifts to older children, who are excluded not only from free healthcare but also from most childhood interventions, such as seasonal chemoprophylaxis for malaria. This lack of consideration for older children raises ethical issues.

When it’s over 5 years, you want it to be 4 or 3 years again. (CG6)

It’s a problem for us, because if they always say it’s those who are under five years old who benefit … they choose some to receive, and others not to receive; that’s always a problem for us. (CG3)

For example, the malaria medicine they give here, if a child is over five years old, they leave and go take another one. But all children are going to get sick from malaria; they should help us with all the children. (CG1)

Another reason associated with circumventing practices is the anticipation of no longer being eligible for free services. In the same vein, some fear that implementation issues, particularly in terms of drug supply, would prevent them from benefiting from free healthcare. This leads some people to accumulate a small stockpile of medicines at home in the event that free care is not always accessible.

You take advantage of the pregnancy and you get treatment. The pregnancy is going to end, where are you going to go to get it fixed? As long as I’m free of this disease, even if it requires to say that it’s only when I became pregnant that the disease came. (CG2)

For example, if my child is sick, I go there to receive the medicine … The problem is when there’s no medicine there. (CG3)

There are people who go from CSPS [health centre] to CSPS. They can go here in the morning to consult, and since it’s close by, they can go to two other health facilities on the same day to collect the products and store them. (HP9)

### Ethical Tensions Experienced by Healthcare Providers

Healthcare providers acknowledge that the situation is not easy for them. On the one hand, they are subject to the hierarchy of the health system and obliged to enforce the rules, in this case, strict compliance with the eligibility criteria for free healthcare. On the other hand, they are moved by the financial difficulties encountered by community members, with whom they share their daily lives. They faced a tension of an ethical and professional nature in their decision to provide free or fee-paying care.

We’re executing personnel of the Ministry of Health, so when it’s a case like that [of circumventing practices], it’s a little bit very difficult, it’s a little bit very difficult. (HP7)

Me, personally, that’s me, I applaud the user fee abolition policy. Why am I praising it today? Because it is when it went free that I understood how poor the people were. (HP5)

In such a context of poverty, many caregivers report their own inability to strictly enforce eligibility criteria, especially among the most vulnerable households.

Sometimes you look at someone, if you see that it’s still not going well, you feel obliged to help, to include the patient in free healthcare so that they can benefit. Some patients, when they come, even five francs [0,01 USD], they don’t have that. (HP1)

A case of malnutrition like this, maybe the child is over 59 months, but physically he/she is not well … . Well, in these cases like this, it happens to bypass, I think in some situation it is legitimate to take appropriate measures. (HP9)

Some also refer to their professional ethics, and justify their decision to extend free healthcare to ineligible patients in view of their commitment to alleviating the suffering of individuals.

The mother insists that her child is five years old, you know the child is over five years old, but you can’t leave the child suffering … and the mother hasn’t brought anything [any money]. (HP10)

Finally, many mentioned their close involvement in the community, and the need to maintain a good relationship with its members. Refusal to provide free care can lead to conflict or fear of having a bad reputation. In some cases, this motivates flexibility on the part of caregivers. This perspective is all the more present as caregivers recognize that circumventing practices are sometimes legitimate, as they are caused by flaws in the system (e.g. stock-outs of medications).

If there’s no notebook, it gets complicated (laughs) … It leads to arguments. Maybe even a fight, if she pretends that it’s her child and that you have to provide care for free, and that’s it! So much so that the health workers will end up treating for free. You have to end up treating her child for free to buy some peace. (CG5)

Imagine, if there’s a drug stock-out here, we say to them that we have to go elsewhere, since I can’t deprive him since he’s eligible for free healthcare … If I give him a prescription so that he can go and buy the medication in a pharmacy, it’s as if the free healthcare policy is not effective in our health area. (HP5)

### Adaptive measures by healthcare providers

Health staff have adopted various informal practices to overcome these tensions. One of them is to systematically demand the health booklet or even the birth certificate of young children, so that their age can be verified. Also, they may give only part of the treatment and require the patient to return several times to take the remaining doses, officially to ensure close monitoring of the patient’s condition. In some cases, the administration of the treatment may even be directly observed.

There are others even who bring the child, they know that the child is over five years old, but in order to take advantage, they will say that the child is under five. If we see that the age of the child will lead to too much discussion, we say to send the child’s birth certificate so that we can have the discussion. (HP1)

Now instead of one box, I give a blister [pack of pills] and schedule an appointment two days later to see if the child has taken the products; sometimes for the antibiotics, it is an 8-day treatment, so instead of giving two boxes, I give one box, I keep the other one here; she takes the first and she comes back later to take the other one. (HP2)

However, healthcare personnel also adopt conciliatory practices. For example, they sometimes use the “ear technique” (i.e. excluding from free admission children who are capable of grasping the opposite ear with one hand while passing their elbow over their head) to facilitate the inclusion of small children in free admission even if they do not have their health booklet. They are also flexible with regard to the cut-off points for eligibility (59th month for children, 42nd day for postpartum), knowing that these have been chosen arbitrarily and are not easily identifiable by the population. They also emphasize raising awareness among mothers about free access, particularly with regard to the dangers of giving medicines to persons other than the patient, the importance of respecting the dosage and of bringing children quickly to a consultation in the event of fever.

You treat these cases as if they were under five. These cases are children who are a few months older than five years, so you tell yourself it doesn’t matter, with the difficult living conditions of the parents in the village, it’s not often easy. (HP10)

Often we find children who are over only by one month, we give care for free […] There is room for manoeuvre. (HP9)

## Discussion

This study shows for the first time that the introduction of a policy of free healthcare raises ethical issues experienced by (non-)beneficiaries and providers, particularly with regard to compliance with eligibility criteria. Circumventing practices illustrate how difficult it is to justify, among the poorest communities, reserving free healthcare only for well-defined sub-categories of the population. While the very delimitation of these criteria, based on the priorities of international organizations, raises ethical issues and reflects power asymmetry, our approach here has rather consisted of exploring the experience of the actors involved in its implementation, at the interface of the patient, the caregiver, and the healthcare provider.

Interviews confirm the presence of some circumventing practices that have been observed in other studies of direct payment exemption policies for healthcare (Druetz et al., 2015; Qiu et al., 2018; Ridde & Diarra, 2009). The mothers’ motivation to employ these practices stems from three factors. The first and most significant explanation relates to the extreme economic vulnerability of some households, which simply do not have the means to cover the remaining fee-paying healthcare services. While some studies have attempted to quantify the money saved per household through free health care (Abdou Illou et al., 2015), it should be noted that the elimination of direct payment does not automatically build up a financial cushion in households, which could then be used to pay for the healthcare of ineligible persons. One cannot set aside resources that one does not have, and it is a mistake to think that free healthcare allows the poorest households to save money for the future.

Second, circumventing practices are associated with low decision-making autonomy of mothers and their lack of control over household resources. These findings corroborate those of a recent systematic review, which highlighted the fact that mothers do not have control over financial resources in the household, and must therefore negotiate with the husband in order to pay the costs associated with healthcare (Beaujoin et al., 2021; Plouffe et al., 2020). Since the burden of caring for children rests mainly on them, mothers try to stretch the criteria for eligibility for free care rather than helplessly witness the progression of disease in their children.

Finally, some participants mentioned a certain disagreement with the eligibility criteria that arbitrarily define time windows of life during which an individual can benefit from healthcare for free. Without understanding why a pre-existing health problem could not be treated free of charge during pregnancy, or why children over the age of five are no longer eligible for most health programmes, they feel justified in relaxing, or even extending, the eligibility criteria. Their concerns echo several studies that have raised the urgency of no longer neglecting the health needs of other vulnerable categories of the population, such as children aged 5–14 years or adolescents (Bhutta et al., 2019; Masquelier et al., 2018). Similarly, several feminist studies have highlighted the way in which the international community has equated women’s health with maternal health and has reduced women to their wombs, particularly in the context of the Millennium Development Goals (Harman, 2012; Thomas, 2003; Tiessen, 2015).

These issues are known to health providers, who perceive the lack of agency of the beneficiaries and are sensitive to the households’ economic vulnerability. This situation places them in an ethical, even deontological dilemma as clinicians: they have a duty to treat and relieve the suffering of patients, but also to ensure that the official guidelines issued by the Ministry of Health are respected. This situation leads them to be flexible, even if it means that they have to be less compliant with the eligibility criteria. Studies have shown the presence of similar practices and dilemmas experienced by clinicians in high-income countries who, in the presence of vulnerable and uninsured patients, modify their reports to make them eligible for exemptions or reimbursements (Hurst et al., 2005; Weiner, 2001). It should be noted, however, that such ethical issues encountered by healthcare workers have rarely been studied in low-income countries, let alone in rural primary healthcare settings (Sippel et al., 2015). Our results suggest that clinicians are confronted with them in an even more blatant manner since they often reside in the community and share the living conditions of its members.

In order to reduce these ethical tensions and to avoid conflicts with the community, clinicians have introduced conciliation practices (close follow-up of patients, directly observed treatment, etc.) which make it possible to limit circumventing practices without being totally inflexible. These modes of reinvention are important to allow the adaptation of the free policy to the local context, promote its acceptability, and optimize its effectiveness under routine implementation conditions on a national scale (Perez et al., 2011). However, the room for reinvention is limited and does not enable them to overcome a major problem associated with the implementation of free healthcare policies, namely the frequent stock-outs of medicines (Hatt et al., 2013). In a mutual cognitive process, these stock-outs justify both the beneficiaries’ circumventing practices and the providers’ flexibility in strictly complying with eligibility criteria.

Ultimately, and although it raises ethical issues, the user fee abolition policy does not raise feelings of injustice or anger in the population. Its imperfections do not prevent the user fee removal policy from receiving the support of beneficiaries and health personnel, despite the increased workload of the latter. Its benefits on maternal and child health are unanimously recognized, which reinforces the expectations of its extension to other target populations (horizontal scale-up) or to other types of care such as family planning (diversification or functional scale-up) (Simmons et al., 2007). Abolishing direct payment in health facilities appears to be the most effective and feasible method of improving financial access to health services. Indeed, experiments of community-based insurance mechanisms proved unsuccessful to reduce out-of-pocket expenditures for health in rural populations in Burkina Faso (Fink et al., 2013). Also, initiatives that removed direct payment only for indigents faced numerous issues and were insufficient to ensure equitable access to healthcare in rural settings (Atchessi et al., 2016; Kadio et al., 2018). Although the cost of such a national policy of free access is not negligible (∼55 million USD in 2018), avenues can be explored to ensure its sustainable financing and improve universal health coverage (Bicaba et al., 2020; Kutzin et al., 2016; Till et al., 2017).

Some limitations of the study should be considered in interpreting the results. First, the study was conducted in a limited number of villages (5), all located in one district. As such, it does not claim to be representative of general perceptions and practices across Burkina Faso. However, the research team has been conducting research on the user fee abolition policy (and, before that, on pilot projects) for many years in Burkina Faso, and there is no indication that the problems related to the respect of eligibility criteria are different in other regions. To the best of our knowledge, the selected communities in the study area are representative of the rural setting in Burkina Faso. A social desirability bias and fear of negative repercussions may have affected the participants’ responses during the interviews, particularly because of the sensitive nature of the subject (circumventing practices can be perceived as fraud) (Miles et al., 2014). To minimize bias, we used an experienced interviewer who speak the local language and was accustomed to dealing with sensitive topics. Also, participants were repeatedly assured of the confidentiality of their responses and the absence of potential negative repercussions.

## Conclusion

Burkina Faso is one of the first countries in sub-Saharan Africa to have introduced a national policy of free healthcare for children under five and pregnant women. While considerably improving access to healthcare for a significant proportion of the population, financial barriers remain for those who are not eligible, which raises ethical issues for caregivers within the most vulnerable households and for providers. This study shows how these individuals have adapted their practices regarding compliance with eligibility criteria, leading to a local reinvention of the free healthcare policy. They also draw attention to the shifting burden of healthcare costs on children aged five years and older, who remain ineligible for many public health interventions.

## Supplementary Material

Supplementary_Material

## Data Availability

All audio recordings can be made available upon reasonable request by contacting the corresponding author.

## Geolocation information

Burkina Faso
