# Supplementary material for: Free healthcare for some, fee-paying for the rest: adaptive practices and ethical issues in rural communities in the district of Boulsa, Burkina Faso
Source: Glob Bioeth. 2021 Aug 13;32(1):100–15. doi: 10.1080/11287462.2021.1966974 (PMC8366671; doi:10.1080/11287462.2021.1966974)
Supplement: Supplementary_Material [file RGBE_A_1966974_SM6993.zip › AF2_Interview_guide v2.docx]

**Adidtional file 2: Interview guides**

**Interview guide for healthcare providers**

**Presentation:** name, title, socio-demographic characteristics, role at the health centre, number of years spent in the community

**Determination of the age of the beneficiaries of free care**

Who are the beneficiaries of free health care?

Who are those who come for free care? Do you know them? How do you know the age of the child?

When you don't know the age of the child, how do you do it?

Is there any flexibility in respecting the criteria at this level concerning the mother or the child? If so, what does this flexibility consist of?

**About ethics**

Are you confronted with ethical issues regarding the strict respect of the instructions related to free care?

How do you live in a practical way this situation of ethics related to free care?

**Perceptions about treatment-seeking practices**

When the mothers come to you, do they come alone?

Do they come for specific questions (ANC for example)?

Does it happen that they come with a child but want treatment for someone else?

**Have you ever observed situations where:**

1. The mother uses her child under the age of 5 years for the purpose of benefiting :

- from care or advice (nutrition, planning, fertility) for herself or for these ineligible grandchildren?
- from consultation fee exemption?

How did you react? How did you resolve this situation?

1. Does the mother use her antenatal visits for care for herself or for her child?

How did you react? How did you resolve this situation?

**Interview guide for caregivers (mothers)**

**Presentation:** age, marital status, socio-demographic characteristics, number of children, number of children under 5, activity.

**Mothers’ knowledge**

Have you heard about free health care in Burkina Faso?

* Do you know who these people are who can benefit from this care for free?

* Do you know what care is covered free of charge?

**The practices of mothers and health workers**

*Care for children*

*What do you do when your child >5 years old is sick? Do you bring him/her to the CSPS? What influences your decision?

*Do you bring the child >5 years old with a younger child to have a consultation of both sets? What happens in this case? Is the consultation free?

*Do you sometimes give the child a free consultation even if he/she is over 5 years old? Is the age really checked at the CSPS? How do the health workers verify the age? And in the other CSPSs around, is it also the same thing?

*Is it a problem that some children get free care, but not others? What do you feel? What to do when a child is sick but does not get free care?

*Does it sometimes happen that free medicines for children < 5 years old can be used for other sick people in the house? What do you mean? Please explain.

Do you know if any other mothers do it? If so, please explain

*Do you sometimes try to use eligible children to get free care for other children, or for yourself? Why do you do that? Do you comply (respect) the official eligibility criteria for free care? How do you feel?

Do you know if other mothers are doing it? If so, explain

*Care for mothers*

* Do you ever go to several CSPS? Why go to more than one CSPS? Do you get more free medicines when you go to several CSPS (for example with a sick child <5 years old)? If so, what is the point of having more free medicines?

* Have you ever observed or heard other mothers doing this? Why do they behave this way?

* When you go for ANC, do you talk about other problems/illnesses with health workers? If yes, what do you talk about? If no, why do you not take advantage of the visit to talk about other problems/illnesses?

* Is it usual to use the child under five years of age to benefit from health services such as family planning?

* Is it usual to have techniques to try to get other (non-eligible) people to benefit from free services? What do you think about this? What do health workers think about this?
