# Supplementary material for: Free healthcare for some, fee-paying for the rest: adaptive practices and ethical issues in rural communities in the district of Boulsa, Burkina Faso
Source: Glob Bioeth. 2021 Aug 13;32(1):100–15. doi: 10.1080/11287462.2021.1966974 (PMC8366671; doi:10.1080/11287462.2021.1966974)
Supplement: Supplementary_Material [file RGBE_A_1966974_SM6993.zip › AF3_Coding_grid v2.docx]

**Additional file 3: Coding grid**

1. Knowledge: Level of knowledge of participants on the existence of free health care, the beneficiaries and the coverage of health care for mothers and children.
2. Beneficiaries (code: Beneficiaries): Participants' knowledge of the beneficiaries covered by the free care
   - Maternal beneficiaries: Mothers' knowledge of the beneficiaries covered by free health care.
   - Beneficiaries’ caregivers : Caregivers' knowledge of the beneficiaries covered by free care
3. Covered Care (Code: Covered Care): Care covered free of charge for mothers and children
   - Mothers (Code: Mother Care)
   - Children (Code: Child Care)
4. Source of information (Code: Information): Source of information for mothers on the existence of free care and the conditions surrounding its implementation.
5. Decision for Fee-Paying Health Care (Code: Paid Health Care : Context of Decision Making for Access to Health Care that Remains Fee-Based for Children Not in Care
6. Family influences: influence related to the family (e.g. authorization of the spouse and influential persons, absence of the father) and which influences the mother's decision to bring her child over five years of age to the health centre (Code: Family influences).
7. Financial influences (code: financial influences): all sources of financing for the care of children outside the target group - Parents' financial inadequacy which disrupts the mother's decision to bring her child over five years old to the health centre ()
8. Autonomous Mothers (Code: Autonomous Mothers): Autonomous decision-making by the mother to bring her child over five years of age to the health centre, even in the absence of the spouse.
9. Practices: Circumventing Practices of mothers to benefit from free care. (Include also those who have never used strategies to make their children benefit from free care, i.e. no practice is a practice).
10. Use of eligible child (code: eligible child): Strategies used by mothers to provide free care to off-target children
    - Child (Code: children children): strategies of mothers using the children eligible for free consultation and medicines to benefit the older ones ;
    - Other (Code: other children): strategies of mothers using children who are eligible for free medication in order to benefit from free medication for the benefit of others.
    - Mothers (Code: child mothers): use of children under five years of age by mothers to benefit from health and care services such as family planning.
    - No practice children (Code: children None): non-use of the child eligible for free out-of-home care
    - Lying age: Practice of mothers who lie about the age of the ineligible child in order to receive free care, or who come for consultation without the ineligible child's record.
11. Use of the mother: Strategies that the beneficiary mothers themselves use to make the benefit free for themselves as well as for other members of the family.
    - Child (Code: Child mothers): Benefit for mothers eligible to receive medicines for older children.
    - Other (Code: Mothers others): Mothers who take advantage of their pregnancy to benefit from the medicines for other family members.
    - For themselves (Code: Mothers Mothers) To use services for themselves, for non-pregnancy care
    - Lack of practice (code: Mother none): Non use of eligible mother for free off-target care
12. Situation (Code: Situation): situation(s) personally experienced by the participants in relation to mothers' circumventing practices.
13. Staff Reaction (Code: Personal Reaction): Reaction of caregivers to get mothers to respect the guidelines of free care or to find a solution that favours mothers in a situation of circumventing practice.
    1. Control practices to support compliance with guidelines: control of caregivers to limit circumventing practices. (Code: Guidelines)
    2. Conciliatory practices in favour of mothers: Practice of reconciling caregivers to find a solution to circumventing practices. (Code: Conciliation)
14. Factors Surrounding Circumventing Practices (Circumventing Factors): Factors that promote or impede circumventing practices care. This does not refer to practices but rather to elements that can lead to (or prevent) circumventing practices.

a. Facilitating factors: Factors facilitating circumventing practices (e.g. distance) (Code: Facilitators)

b. Barriers (Code: Barriers): Factors that Disadvantage circumventing practices

1. Perceptions (code: perception): participants' perception of the advantages and weaknesses of free care.
   1. Advantages: Advantages/strengths of free health care reported by the : (Code: Advantages)
      - Mothers (Code: Parental Benefits)
      - Caregiver (Code: Caregiver Benefits)
   2. Weaknesses: Weaknesses of the gratuity policy reported by (Code: weaknesses)
      - Mothers (Code: Weak Mothers)
      - Caregivers (Code: Caregiver Weaknesses)
2. Ethical, moral, or deontological issues (code: ethics): Ethical and moral tensions, constraints linked to what the participants feel about the ethical issues of free care (*tiring, difficult to tell a mother to go to another CSPS to look for products that cost 1000 CFA*).
   1. Reported by Mothers (Code: Mother Ethics)
   2. Reported by Caregivers (Code: Caregiver Ethics)
3. Emerging Themes: Themes not considered in the current codification but emerging in the interviews. (Code: Emerging)
   1. Membership (code: accession): Accession of women/spouses to family planning
   2. Refusal (Code: Refusal): Spouses' non-adherence to family planning
